# Supplementary material for: Deletion of wfs1 Impairs Oligodendrocyte Precursor Cells Dorsal Distribution and Myelination Through the wfs1-hmgcs1 Axis in Zebrafish
Source: Biology (Basel). 2026 Aug 16;15(16):1402. doi: 10.3390/biology15161402 (PMC13510151; doi:10.3390/biology15161402)
Supplement: Supplementary file 1 [file biology-15-01402-s001.zip › File S1-Transcriptome sequencing data and differentially expressed genes (DEGs) in wfs1-- zebrafish at 5 dpf/2.KEGG/wfs1KOvsWT/ALL/src/dre03010.html]

dre03010


- dre:550564

- Down regulated genes

550564\_1(-1.62314729124795)

- dre:337007

- Up regulated genes

337007\_1(2.20587736514709)

- Down regulated genes

337007(-1.672701928124)

- dre:327089

- Up regulated genes

327089(3.97047596560987)

- dre:560828

- Up regulated genes

560828\_1(3.03603892081494)

- dre:415196

- Up regulated genes

415196\_1(6.18252739825696)

- dre:768187

- Down regulated genes

768187\_1(-1.96198384996488)

- dre:553799

- Down regulated genes

553799(-1.25422339743704)

- dre:336712

- Up regulated genes

336712\_2(8.32782168527236)

- Down regulated genes

336712(-1.23320552363898)

- dre:554098

- Down regulated genes

554098\_2(-7.30228637661142)

- dre:450009

- Down regulated genes

450009\_1(-1.31598924929418)

- dre:553284

- Up regulated genes

553284\_1(1.53871042189345)

- Down regulated genes

553284(-1.44283750129257)

Close
